# The Dependence of Implicit Solvent Model Parameters and Electronic Absorption Spectra and Photoinduced Charge Transfer

**DOI:** 10.1038/s41598-020-60757-1

**Published:** 2020-02-28

**Authors:** Xiangtao Chen, Wenhua Qiao, Wenjing Miao, Yangdong Zhang, Xijiao Mu, Jingang Wang

**Affiliations:** 1 Computational Center for Property and Modification on Nanomaterials, College of Sciences, Liaoning Shihua University, Fushun, 113001 P.R. China; 2 School of Mathematics and Physics, University of Science and Technology Beijing, Beijing, 100083 P.R. China; 3 Liaoning Huadian Tieling Power Generation Co., Ltd., Tieling, 112000 P.R. China

**Keywords:** Electron transfer, Computational science

## Abstract

In this work, the relationship between multiple solvent parameters and charge transfer index was analyzed by multi-factor multi-variate partial least squares regression (PLSR). The charge transfer of the molecule is visualized by the analysis of the excited state wave function. Hydrogen bond basicity and surface tension can significantly affect charge transfer by studying the solvation model parameters and charge transfer index. Finally, a method in which a solvent regulates charge transfer strength and migration length is proposed.

## Introduction

Photoinduced charge transfer is the act of transferring electrons in a molecule away from the original position to other atoms when the molecule is excited by light^1^. It is widely found in conjugate systems and donor-acceptor systems^2^. This special charge transfer behavior has a good application prospect in the fields of photocatalysis^3,4^, biophotonics^5,6^ and solar cells^7,8^. There have been many studies on charge transfer and the nature of molecules themselves, such as conjugation^2^, push-pull electrons^9,10^, and electronegativity^11^. It has also been suggested that the addition of an external electric field and the charge such as can significantly improve the charge transfer efficiency. However, there are few studies on the charge transfer of solvents. In particular, various parameters of the solvent have little research on the intensity of charge transfer and the migration distance^12,13^.

The implicit solvent model does not specifically describe the specific structure and distribution of solvent molecules in the vicinity of the solute, but rather considers the solvent environment simply as a polarizable continuum^14,15^. The advantage of considering the solvent effect is that it can express the average effect of the solvent without the need to consider the arrangement of various possible solvent layer molecules as in the explicit solvent model, and it does not increase the computational time and therefore is high. Widely used in the field of quantum chemistry and molecular simulation.

The implicit solvent model will change the potential energy surface of the system. Therefore, the direct correlation with the potential energy surface, such as single point energy, minimum point and transition state structure, vibration frequency, different conformation distribution ratio, excitation energy, etc. will also be affected^16–19^. The implicit solvent model also affects the electronic structure of the system, so the properties of gap, dipole moment, bond level, atomic charge, etc. are also affected^20^.

Some properties of the system are greatly influenced by the implicit solvent model, such as excitation energy, HOMO-LUMO gap, dipole moment, atomic charge; some are affected little, such as geometric structure, vibration frequency^21^. But here is only most of the situation, specifically depends on the actual system. In addition, the greater the polarity of the solute and solvent, the stronger the electrostatic interaction and the more pronounced the solvent effect.

In this work, the relationship between multiple solvent parameters and charge transfer index was analyzed by multi-factor multivariate partial least squares regression (PLSR)^22^. A quantitative analysis of the relationship between different factors and charge transfer was performed. A method in which a solvent regulates charge transfer strength and migration length is proposed.

## Method

**Figure 1:**
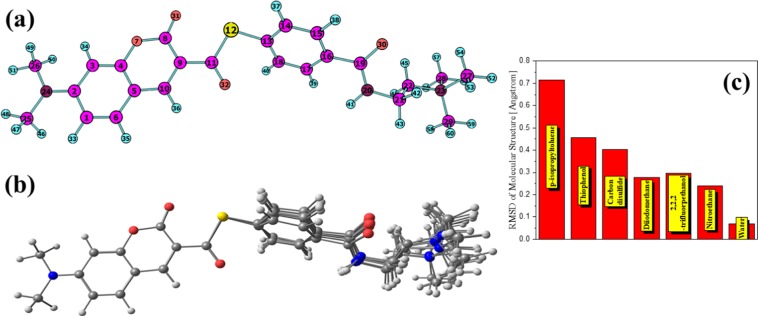
(**a**) Molecular structure and atomic number, (**b**) merge image of molecular structure in different solvents. (**c**) The RMSD values analysis histogram of molecular structure referred by the molecule without solvent.

The molecular structure (see Fig. 1) are optimized with density functional theory (DFT)^23^ B3LYP^24^ functional, and 6–31 g(d) basis set and the cartesian coordinate of optimized structure are shown in the Tables S1~S8 in Supporting Information. To more accurately describe the nature of electron long-range transfer during excitation, the electronic transitions of molecule are calculated with time-dependent DFT (TD-DFT)^25,26^, CAM-B3LYP^27^ functional, and 6–31 g (d) basis set. All the quantum calculations are performed with Gaussian 16 software^28^. The transition density matrix (TDM) and electron hole pairs analysis and the transition dipole moment density matrix are performed by the Multiwfn 3.6 program^29^. Density maps in 3D space are drawn using VMD software^30^.

In this work, the molecular excitation behavior is less affected by the solvent. Therefore, in order to more intuitively analyze the effect of the implicit solvent model on charge transfer, a series of indices related to charge transfer are calculated, which is also to quantitatively study the change of charge transfer. The first is the charge transfer length index, which is defined by the electron and hole density:

$$\begin{array}{c}{\sigma }_{{\rm{holex}}}=\sqrt{\int {(x-{X}_{{\rm{hole}}})}^{2}{\rho }^{{\rm{hole}}}\,({\bf{r}})d{\bf{r}}}\\ \Delta {\sigma }_{\lambda }={\sigma }_{{\rm{ele}},\lambda }-{\sigma }_{{\rm{hole}}\lambda }\,\lambda =\{x,y,z\}\end{array}$$

where X is the cartesian component of electron or hole density. The ${\sigma }_{{\rm{holex}}}$ is the RMSD of hole. Therefore, the $\Delta {\sigma }_{\lambda }$ is the RMSD difference of electron-hole pair density. From the above index, two indices of charge transfer can also be defined.

$$\begin{array}{rcl}{H}_{\lambda } & = & ({\sigma }_{\text{ele},\lambda }+{\sigma }_{{\rm{hole}}\lambda })/2\,\lambda =\{x,y,z\}\\ {H}_{{\rm{CT}}} & = & |{\bf{H}}\cdot {{\bf{u}}}_{{\rm{CT}}}|\\ H\,{\rm{index}} & = & (|{\sigma }_{{\rm{ele}}}|+|{\sigma }_{{\rm{hole}}})/2\\ t\,{\rm{index}} & = & {D}_{{\rm{index}}}-{H}_{{\rm{CT}}}\end{array}$$

where the ${{\bf{u}}}_{{\rm{CT}}}$ is the transition dipole moment of charge transfer and the ${D}_{{\rm{index}}}$ is the total magnitude of CT length, which is defined by:

$$\begin{array}{rcl}{D}_{{\rm{x}}} & = & |{X}_{{\rm{ele}}}-{X}_{{\rm{hole}}}|\,{D}_{{\rm{y}}}=|{Y}_{{\rm{ele}}}-{Y}_{{\rm{hole}}}|\,{D}_{{\rm{z}}}=|{Z}_{{\rm{ele}}}-{Z}_{{\rm{hole}}}|\\ D\,{\rm{index}} & = & |{\bf{D}}|\equiv \sqrt{{({D}_{x})}^{2}+{({D}_{y})}^{2}+{({D}_{z})}^{2}}\end{array}$$

where the X, Y, Z is the cartesian component of electron or hole density centroid position. Finally, the charge transfer length is defined as:

$$\Delta {r}_{i}^{a}=\frac{{({K}_{i}^{a})}^{2}}{\sum _{i,a}{({K}_{i}^{a})}^{2}}|\langle {\varphi }_{a}|{\bf{r}}|{\varphi }_{a}\rangle -\langle {\varphi }_{i}|{\bf{r}}|{\varphi }_{i}\rangle |$$

where the ${K}_{i}^{a}$ is the configuration coefficient corresponding to excitation from i to a. The index I and a run over all occupied and virtual MOs, respectively. The ${\varphi }_{a}$ represent the wave function of excited states.

## Result and Discussion

In this work, we calculate the electronic excited spectra, transition density matrix and others electronic excitation parameters for the molecule of S-(4-((2-(dimethylamino)ethyl)carbamoyl)phenyl)6-(dimethylamino)-2-oxo-2H-chromene-3-carbothioate in different solvent, which solvent model parameters have been reported in the literature. The molecular structure is shown in Fig. 1(a). These solvent model parameters are dynamic and static dielectric constant, hydrogen bond acidity/basicity, surface tension at interface, carbon aromaticity and electronegative halogenicity. In order to explore the influence of solvent parameters on the electron excitation in a wider range, the differences between the parameters of our chosen solvents are as large as possible. Although the pseudo-solvent of each parameter can be completely customized, the results discussed in this case may not be well applied in the experiment. Therefore, we selected these solvents in Table 1. The SMD^14^ model parameters for different solvents are shown in Table 1. The first four solvents are p-isopropyltoluene, Thiophenol, Carbon disulfide and Diiodomethane. These solvents have a relatively small dynamic dielectric constant (Eps) and are non-polar solvents. The latter three are 2,2,2-trifluorpethanol, Nitroethane and water. These solvents have a large dynamic dielectric constant and are non-polar solvents. Firstly, the molecular structure in different solvents is optimized. As shown in Fig. 1(b), the change in molecular structure in the different solvents is mainly concentrated on the right side of the figure. That is to say, the left side of the molecule changes little. Other solvation parameters vary less but may effect on electronic excitation.

**Table 1:** SMD model parameters for different solvents.

Solvent model parameters	p-isopropyltoluene	Thiophenol	Carbon disulfide	Diiodomethane	2,2,2-trifluorpethanol	Nitroethane	Water
Eps	2.23	4.27	2.61	5.32	26.72	28.29	78.35
Eps(infinity)	2.20	2.52	2.66	3.03	1.66	1.93	1.77
Hydrogen bond acidity	0	0.09	0	0.05	0.57	0.02	0
Hydrogen bond basicity	0.19	0.16	0.07	0.23	0.25	0.33	0
Surface tension	38.34	55.24	45.45	95.25	42.02	46.25	0
Carbon aromaticity	0.6	0.857	0	0	0	0	0
Electronegative halogenicity	0	0	0	0	0.5	0	0

**Figure 2:**
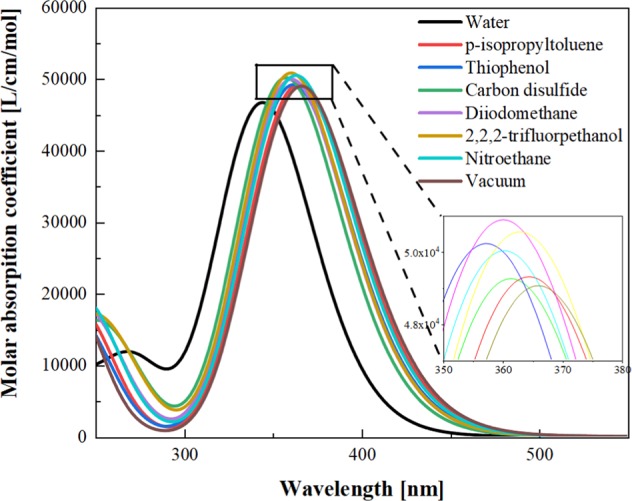
Absorption spectra of molecules in different solvents, the insert image is the partial enlarged view.

To study the effect of solvation parameters on the electron excitation, the electron excitation absorption spectrum was calculated. As shown in Fig. 2, all of electronic absorption spectra are calculated by DFT. In the TDDFT calculation, for all solvent model configurations, we calculated 50 excited states, and used the “IOp” keyword to output all molecular orbitals configuration coefficients. However, because molecular orbitals are dispersed throughout the molecular space, it is necessary to perform electron-hole pair analysis by combining configuration coefficients^2^. The black curve in the electronic spectrum, that is, the absorption spectrum peak in the aqueous solvent is on the short wavelength side. After switching to different solvent parameters, it was found that the spectral peaks had a certain degree of red shift. However, the difference between the different spectra is small. From the illustration in Fig. 2, there is still a big difference between the denser spectra. The dielectric constant of the solvent that absorbs the peak of the spectral spectrum is not correlated.

**Figure 3:**
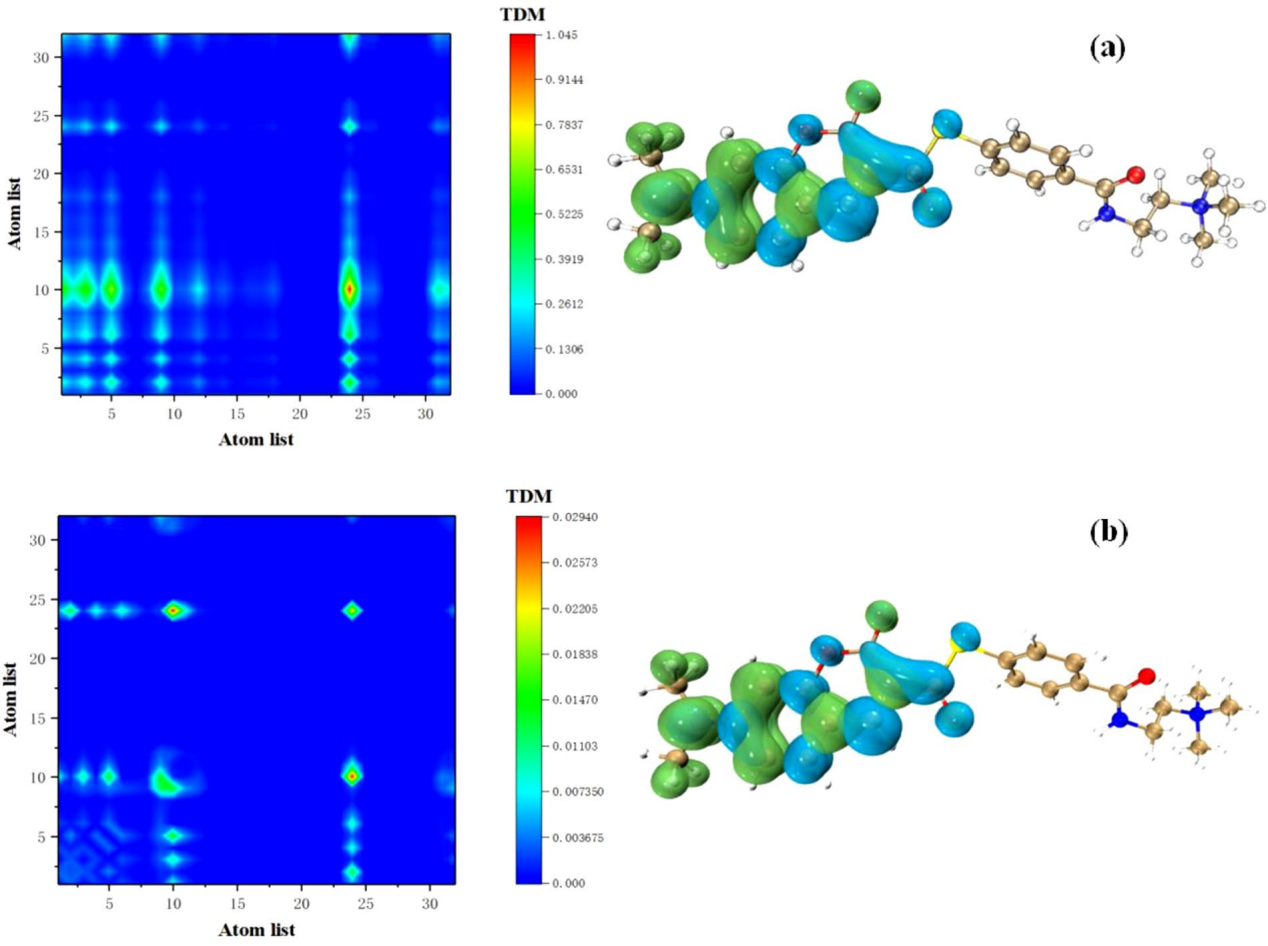
S_1_ TDM and electron-hole pair analysis of molecule in 2,2,2-trifluorpethanol (**a**) and Nitroethane (**b**).

Therefore, first consider the electronic excitation characteristics of this absorption peak. TDM and electron-hole pair analysis of the first excited state of the molecule is shown in Figs. 3 and S1 in the supporting information. In general, the isosurface map of the electron-hole pair analysis is very small, but significant changes can be seen in the TDM diagram. Although it appears that the charge transfer is similar during the electronic excitation process. Electron excitation is concentrated on the left side of the molecule, and there is almost no transition density on the right side. However, charge transfer has always existed. All charge transfer is present from the middle of the molecule to the left side of the molecule. However, as shown in the TDM diagram in Fig. 3(a), the excited state characteristics are quite different from those in other solvents. Mainly manifested as the increase in non-diagonal elements in TDM and enhanced charge transfer. This is the electronic excitation characteristic of the molecule in diiodomethane. With the phenomenological analysis of the solvent parameters, the surface tension of this solvent is very large, reaching 95.25 kcal/mol. Therefore, it can be said that the surface tension of the solvent has a great influence on the charge transfer.

**Figure 4:**
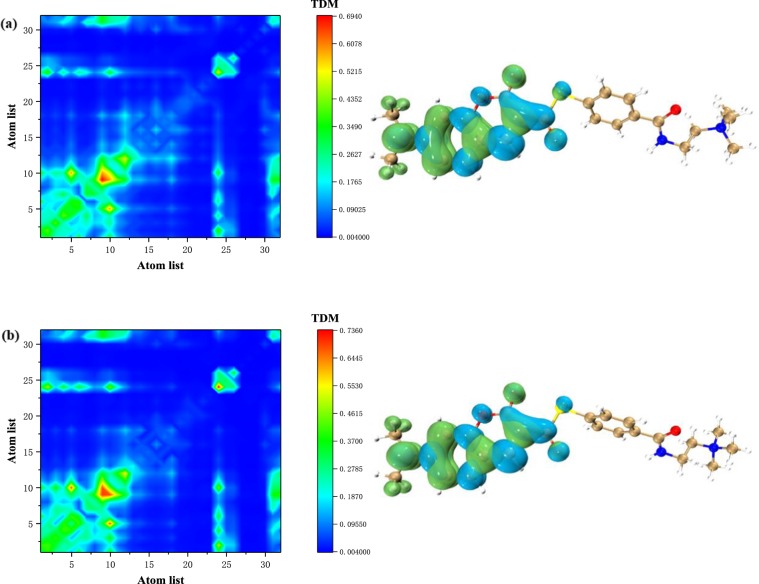
TDM and electron-hole pair analysis of molecule in Water (**a**) and Vaccum (**b**).

To compare with the solvated electron excitation characteristics, the electron excitation spectrum, TDM and electron hole density in water solvent and vacuum were analyzed. As shown in Fig. 4, the results of the electronic excitation analysis of the molecules under water and vacuum, respectively. It can be found that the local excitation on the benzene ring on the left side of the molecule is significantly enhanced in both cases. Although charge transfer still exists, the local excitation portion has already played a major role. The dynamic dielectric constant of water is very large, reaching 78.35, which is a typical polar solvent, and the static dielectric constant is not much different from other solvents. It can therefore be concluded that a high dynamic dielectric constant results in enhanced local excitation of the molecule. Combined with previous conclusions, there is a positive correlation between surface tension and charge transfer.

**Figure 5:**
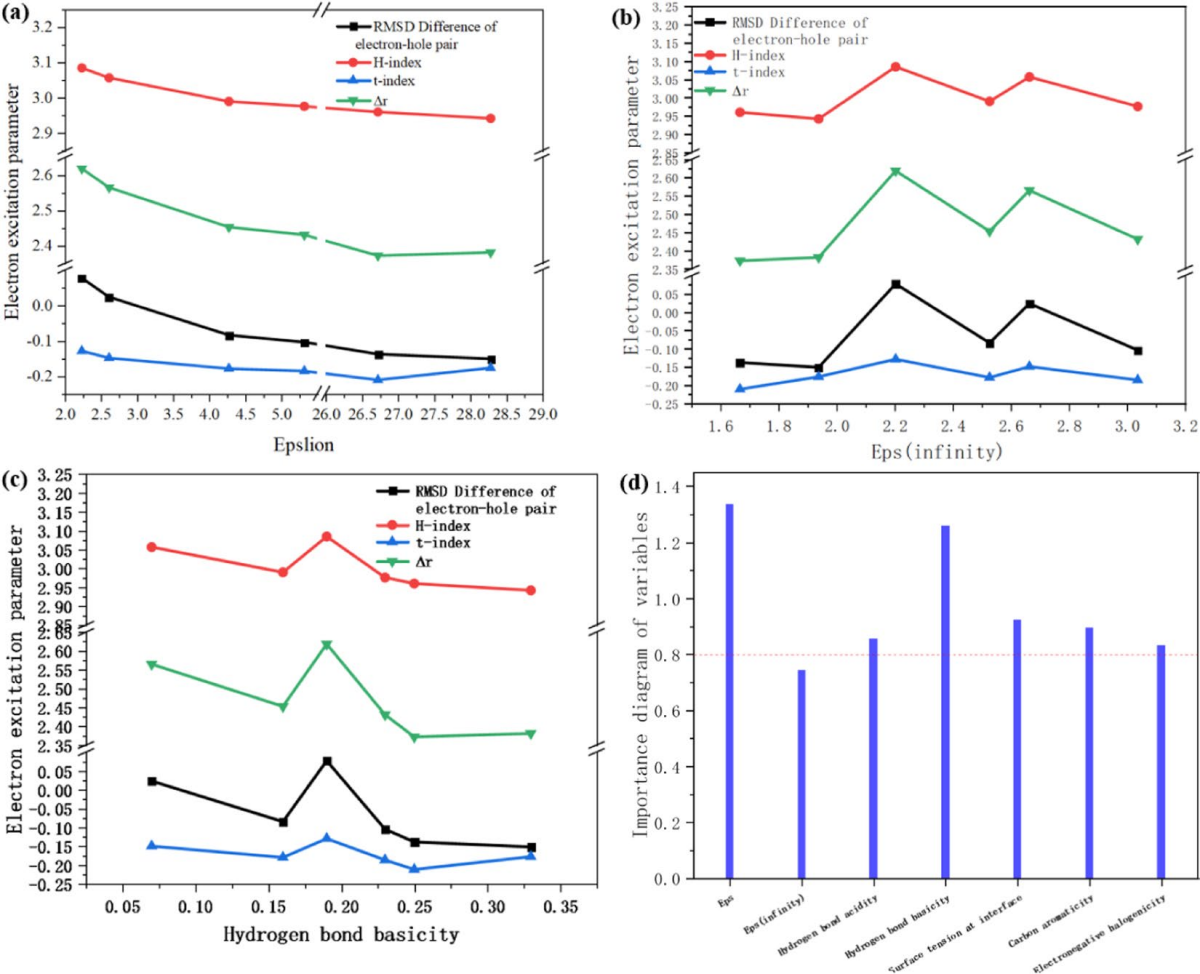
The relationship between electronic excitation parameters and dynamic dielectric constant, static dielectric constant and hydrogen bond basicity; the importance of solvation parameters for electronic excitation characteristic parameters by PLSR.

The charge transfer excitation indices in different solvents were calculated and recorded in Table 2. The charge transfer excitation indices in different solvents were calculated and recorded in Table 2. The change of D index and H index is relatively large, so the details that cannot be detected in the visualization method can be seen through the index. As shown in Fig. 5(a–c), is a line chart between the three solvation parameters and the charge transfer index. It can be seen from Fig. 5(a) that as the dynamic dielectric constant increases, the four charge transfer indices tend to decrease. The relationship between the static dielectric constant and the four indices is not unique. The four indices showed a maximum peak when the Hydrogen bond basicity was around 0.2, indicating that Hydrogen bond basicity effect on charge transfer.

**Table 2:** The relationship of SMD model parameters for different solvents and electronic excitation index.

Solvent model parameters	p-isopropyltoluene	Thiophenol	Carbon disulfide	Diiodomethane	2,2,2-trifluorpethanol	Nitroethane	Water
Excited Energy (eV)	3.40	3.43	3.47	3.44	3.44	3.41	3.60
Oscillator Strength	1.2173	1.216	1.2401	1.2351	1.2564	1.2482	1.1533
D index	2.371	2.380	2.618	2.452	2.565	2.43	1.588
H index	2.960	2.942	3.085	2.99	3.057	2.976	3.16
t index	−0.212	−0.178	−0.130	−0.18	−0.150	−0.187	−1.22
Delta_r	2.371448	2.380514	2.618195	2.452351	2.564824	2.430539	4.93183

Both the solvation parameters and the charge transfer index are multifactorial parameters. In other words, multiple solvation parameters effect on molecular excitation, and these parameters have a large or small effect on the electron excitation or charge transfer index, which is positive and negative. Therefore, it is not accurate to analyze one of the relationships separately. Ordinary multivariate linear regression is often subject to many limitations. The most typical problem is the multiple correlation between independent variables. And sometimes there are very few examples, even less than the dimensions of variables, and there are multiple correlations between variables. Therefore, partial least squares regression (PLSR) is born to solve these thorny problems. The importance of the solvation parameters was first calculated by the PLSR method, see Fig. 5(d). It can be seen from the figure that the solvation parameter that has the greatest influence on electron excitation and charge transfer is the dynamic dielectric constant, followed by Hydrogen bond basicity, followed by surface tension. The least affected is the static dielectric constant. This result is consistent with the previous discussion. Therefore, these analyses can be used to determine that charge transfer is related to dynamic dielectric constant, Hydrogen bond basicity and surface tension.

To further discuss the correlation between solvation parameters and charge transfer index, the regression coefficients for the PLSR method are listed in Table 3, where the first row is the constant term in the regression. It can be found that there is a negative correlation between the degree of charge transfer, that is, the H factor and the dynamic dielectric constant, and a positive correlation with Hydrogen bond basicity. For example, there is a negative correlation between aromaticity and H factor. For the RMSD of electrons and holes, the relationship between the dynamic dielectric constant and the dynamic dielectric constant is exactly the opposite, which is also common sense because of the different electrical properties. The electronegative halogenicity has a very large coefficient of RMSD for electrons, which also represents the solvent’s push-pull effect on electrons in the molecule. On the other hand, for the charge transfer length, that is, the delta_r parameter, the influence of Hydrogen bond acidity and Electrogenegative halogenicity is large. Therefore, solvents can be used to regulate charge transfer.

**Table 3:** Linear fitting parameters of solvation parameters for electronic excitation characteristic parameters by PLSR.

Regression coefficient	D index	RMSD of hole	RMSD of electron	H index	t index	Delta_r
Intercept	2.788196	3.127682	3.615751	3.058753	−0.30283	3.053481
Eps	−0.01694	0.001236	−0.00404	−0.01601	−0.01491	−0.00447
Eps(infinity)	−0.00496	−0.04978	−0.25717	0.10404	0.181859	−0.22466
Hydrogen bond acidity	0.324084	−0.54769	−2.50306	1.428786	2.241504	−2.19652
Hydrogen bond basicity	0.93852	−0.33147	−0.92203	1.342404	1.728784	−0.73725
Surface tension at interface	−0.00509	0.001559	0.004691	−0.00728	−0.0091	0.003805
Carbon aromaticity	−0.17421	0.048978	0.131577	−0.23829	−0.28996	0.106101
Electronegative halogenicity	−0.32309	0.561513	2.541562	−1.37621	−2.28215	2.176552

## Conclusion

In this work, the electron absorption spectra and charge transfer indices of the same molecules in different solvents were calculated by DFT method, and the quantitative analysis of the effects of various solvent parameters on charge transfer was performed by PLSR method. High dynamic dielectric constant, hydrogen bond basicity and surface tension can effectively affect charge transfer behavior. Although it is an indisputable fact that the solvation effect can regulate the charge transfer behavior, the effects of various characteristic parameters of the solvent on the electronic excitation and charge transfer can be studied theoretically more precisely and quantitatively through PLSR analysis. Hydrogen bond acidity/basicity are a pair of opposite parameters. This pair of parameters is almost opposite for H index, the cavity RMSD. However, the impact on electronic RMSD varies greatly. For various charge transfer parameters, the influence of static permittivity is greater than that of dynamic permittivity. This regulation can both regulate the charge transfer intensity and regulate the charge transfer length. It has different meanings in different application scenarios. This will be a very important rule for designing a solvent or selecting a suitable solvent. This law can be well applied in the fields of photocatalysis, biophotonics and solar cells. And in the experimental measurement of the absorption spectrum, it is also very important for theoretical interpretation and error analysis.

## Supplementary information

Supplementary information
