# Supplementary material for: The Dependence of Implicit Solvent Model Parameters and Electronic Absorption Spectra and Photoinduced Charge Transfer
Source: Sci Rep. 2020 Feb 28;10:3713. doi: 10.1038/s41598-020-60757-1 (PMC7048810; doi:10.1038/s41598-020-60757-1)
Supplement: Supplementary file 1 — Supplementary information [file 41598_2020_60757_MOESM1_ESM.pdf]

Supporting Information

The Dependence of Implicit Solvent Model Parameters and Electronic Absorption Spectra and Photoinduced Charge Transfer

Xiangtao Chen,[1,†] Wenhua Qiao,[1,2,†] Wenjing Miao,[1,†] Yangdong Zhang,[3] Xijiao Mu,[2,]* Jingang Wang[1,]*

[1] Computational Center for Property and Modification on Nanomaterials, College of Sciences, Liaoning Shihua University, Fushun 113001, PR China;

[2] School of Mathematics and Physics, University of Science and Technology Beijing, Beijing 100083, PR China;

[3] Liaoning Huadian Tieling Power Generation Co., Ltd. Tieling 112000, PR China;

* CORRESPONDING AUTHORS. EMAIL: muxijiao@gmail.com **(X. J. Mu)**; jingang_wang@lnpu.edu.cn **(J. G. Wang)**

†: Contributed equally

Table S1. The cartesian coordinates of molecule in the p-isopropyltoluene solvent

Elements	X	Y	Z	Elements	X	Y	Z
**C**	7.173525	2.101852	0.386759	O	3.350033	-2.644203	-0.662693
**C**	7.903067	0.933971	-0.001625	O	0.934618	0.609884	0.441360
**C**	7.177380	-0.246793	-0.279367	H	7.701698	3.019768	0.606372
**C**	5.806713	-0.237170	-0.168728	H	7.666467	-1.163010	-0.579811
**C**	5.074595	0.902055	0.212403	H	5.271838	2.971550	0.783465
**C**	5.810927	2.076709	0.487203	H	3.090243	1.660631	0.580930
**O**	5.151656	-1.402715	-0.448107	H	-1.259961	-2.687408	1.710365
**C**	3.786367	-1.543190	-0.383029	H	-3.638588	-2.076532	2.026097
**C**	3.022144	-0.379643	0.005191	H	-3.381512	0.616242	-1.310445
**C**	3.677291	0.794624	0.289833	H	-1.037791	-0.015421	-1.646503
**C**	1.555330	-0.386461	0.113438	H	-4.893660	1.536808	-0.126075
**S**	0.686688	-1.922960	-0.259606	H	-7.226673	0.919576	1.464116
**C**	-0.992436	-1.381294	0.018972	H	-6.940584	2.361886	0.474120
**C**	-1.726758	-1.963940	1.049991	H	-7.519157	1.124503	-1.589014
**C**	-3.059179	-1.619308	1.231610	H	-7.650263	-0.394699	-0.676379
**C**	-3.661835	-0.672619	0.405667	H	9.695544	2.992982	-0.497898
**C**	-2.923612	-0.093978	-0.629272	H	9.846582	2.517173	1.208922
**C**	-1.598900	-0.453755	-0.828173	H	11.054833	1.991995	0.028549
**C**	-5.099657	-0.347467	0.659554	H	9.798103	-1.059634	0.218019
**N**	-5.540479	0.863510	0.260015	H	9.687607	-0.577514	-1.489263
**C**	-6.914545	1.272178	0.479436	H	11.044438	-0.025417	-0.495006
**C**	-7.791084	0.686124	-0.627307	H	-10.880503	0.297125	0.757684
**N**	-9.274914	0.908869	-0.462235	H	-9.383259	0.662556	1.646765
**N**	9.251840	0.959795	-0.100028	H	-9.506483	-0.846527	0.692613
**C**	9.992987	2.182445	0.176172	H	-11.030759	0.483518	-1.556597
**C**	9.977124	-0.240661	-0.487459	H	-9.596786	0.869972	-2.553336
**C**	-9.794766	0.203050	0.750140	H	-9.718492	-0.718487	-1.737939
**C**	-9.955953	0.343093	-1.669254	H	-9.172497	2.872298	-1.238289
**C**	-9.592789	2.366389	-0.368765	H	-10.676811	2.479900	-0.353694
**O**	-5.847796	-1.152825	1.223400	H	-9.169405	2.769922	0.549637

Table S2. The cartesian coordinates of molecule in the thiophenol solvent

Elements	X	Y	Z	Elements	X	Y	Z
**C**	7.193498	2.093614	0.384284	O	3.345433	-2.637141	-0.646305
**C**	7.916141	0.924317	-0.005856	O	0.946149	0.629535	0.459291
**C**	7.185109	-0.251858	-0.27918	H	7.724713	3.01009	0.600122
**C**	5.812348	-0.240334	-0.164529	H	7.668626	-1.170943	-0.577797
**C**	5.088943	0.902895	0.217878	H	5.297961	2.971707	0.785984
**C**	5.829999	2.073289	0.48912	H	3.097383	1.662596	0.590846
**O**	5.156433	-1.402193	-0.440149	H	-1.284458	-2.731463	1.658004
**C**	3.789127	-1.542452	-0.371983	H	-3.676807	-2.130641	1.957315
**C**	3.030084	-0.369862	0.018236	H	-3.365695	0.667023	-1.280183
**C**	3.687485	0.799046	0.299547	H	-1.013638	0.053075	-1.595922
**C**	1.558086	-0.366168	0.130096	H	-4.894844	1.555875	-0.096393
**S**	0.687099	-1.905003	-0.250614	H	-7.210919	0.916701	1.467676
**C**	-0.993038	-1.367377	0.020576	H	-6.954853	2.357531	0.463796
**C**	-1.744189	-1.985416	1.018751	H	-7.529341	1.056407	-1.586914
**C**	-3.079428	-1.647999	1.192419	H	-7.671278	-0.422886	-0.616008
**C**	-3.669844	-0.673502	0.391004	H	9.728907	2.977769	-0.492413
**C**	-2.915946	-0.060418	-0.612283	H	9.859306	2.489914	1.21288
**C**	-1.587974	-0.411824	-0.802892	H	11.075137	1.964446	0.042465
**C**	-5.111348	-0.357102	0.636317	H	9.827114	-1.071329	0.19766
**N**	-5.550557	0.863561	0.231831	H	9.671286	-0.590735	-1.506694
**C**	-6.918561	1.268421	0.476355	H	11.051027	-0.034447	-0.549553
**C**	-7.807196	0.658964	-0.609879	H	-10.892647	0.380992	0.822476
**N**	-9.291248	0.902468	-0.453685	H	-9.378707	0.777723	1.666377
**N**	9.267576	0.945955	-0.110656	H	-9.536291	-0.780994	0.807061
**C**	10.013446	2.161032	0.179628	H	-11.05385	0.435934	-1.529398
**C**	9.985371	-0.252539	-0.51309	H	-9.613134	0.734153	-2.54106
**C**	-9.809101	0.273588	0.80409	H	-9.76294	-0.795015	-1.628974
**C**	-9.981498	0.271972	-1.626103	H	-9.179418	2.819869	-1.343941
**C**	-9.597301	2.368301	-0.444781	H	-10.679038	2.494357	-0.430591
**O**	-5.86772	-1.157324	1.18096	H	-9.165202	2.823911	0.443846

Table S3. The cartesian coordinates of molecule in the carbon disulfide solvent

Elements	X	Y	Z	Elements	X	Y	Z
**C**	7.286280	2.003627	0.270253	O	3.246688	-2.623935	-0.442885
**C**	7.962462	0.771925	0.020954	O	0.972985	0.838951	0.217737
**C**	7.185880	-0.389110	-0.165235	H	7.853848	2.911731	0.419146
**C**	5.810523	-0.307063	-0.105007	H	7.631410	-1.354888	-0.355565
**C**	5.132505	0.899715	0.136812	H	5.422435	3.000767	0.514084
**C**	5.919400	2.054346	0.323657	H	3.161427	1.779874	0.352914
**O**	5.113789	-1.459639	-0.292328	H	-1.453675	-2.544845	1.596372
**C**	3.737317	-1.534661	-0.261469	H	-3.845616	-1.890986	1.775349
**C**	3.022840	-0.291740	-0.016922	H	-3.277183	0.967063	-1.375194
**C**	3.722023	0.867379	0.172295	H	-0.928668	0.303743	-1.566746
**C**	1.547489	-0.214870	0.039242	H	-4.878619	1.871060	-0.290678
**S**	0.632250	-1.760042	-0.170456	H	-7.181604	1.380962	1.219615
**C**	-1.034885	-1.154271	0.011723	H	-7.009729	2.637107	-0.014470
**C**	-1.859281	-1.773742	0.950239	H	-7.947566	1.273825	-1.732010
**C**	-3.193164	-1.407719	1.057261	H	-7.431238	-0.219725	-0.925292
**C**	-3.711709	-0.400437	0.246527	H	9.883785	2.686975	-0.587242
**C**	-2.885052	0.215748	-0.696838	H	9.946078	2.354908	1.158740
**C**	-1.558588	-0.164202	-0.820656	H	11.166974	1.670746	0.079161
**C**	-5.148350	-0.050549	0.414648	H	9.765247	-1.279426	0.488950
**N**	-5.560590	1.163946	-0.062268	H	9.696124	-0.962126	-1.258633
**C**	-6.929518	1.565372	0.171799	H	11.065922	-0.379044	-0.303503
**C**	-7.849520	0.772323	-0.767709	H	-10.285738	-0.570494	1.209493
**N**	-9.256281	0.549975	-0.256448	H	-8.798046	0.211892	1.798301
**N**	9.319658	0.721786	-0.035350	H	-8.673088	-1.218246	0.757676
**C**	10.111453	1.923586	0.165133	H	-11.048522	-0.311364	-0.993051
**C**	9.989460	-0.541726	-0.290024	H	-10.023822	0.466232	-2.231778
**C**	-9.252924	-0.321361	0.967673	H	-9.548806	-1.110637	-1.538769
**C**	-10.026186	-0.152404	-1.334631	H	-9.922005	2.470460	-0.851195
**C**	-9.920106	1.855467	0.049021	H	-10.942839	1.663715	0.372234
**O**	-5.959318	-0.806909	0.943720	H	-9.375246	2.359900	0.845437

Table S4. The cartesian coordinates of molecule in the diiodomethane solvent

Elements	X	Y	Z	Elements	X	Y	Z
**C**	7.239012	2.065006	0.280949	O	3.313477	-2.647443	-0.511085
**C**	7.946062	0.849638	0.032909	O	0.956357	0.754508	0.192481
**C**	7.197425	-0.327833	-0.171339	H	7.784604	2.984637	0.440363
**C**	5.820644	-0.275934	-0.125197	H	7.667757	-1.281320	-0.364365
**C**	5.112626	0.913154	0.119436	H	5.350755	3.018386	0.511475
**C**	5.871356	2.084910	0.320928	H	3.123447	1.745790	0.334933
**O**	5.149534	-1.442181	-0.329298	H	-1.327522	-2.522412	1.710541
**C**	3.775883	-1.547223	-0.310455	H	-3.717030	-1.878040	1.947361
**C**	3.031743	-0.326126	-0.054759	H	-3.339583	0.705304	-1.457572
**C**	3.704550	0.847843	0.147334	H	-0.990442	0.051884	-1.704131
**C**	1.555719	-0.283891	0.003449	H	-4.888482	1.682491	-0.367195
**S**	0.670816	-1.845368	-0.214590	H	-7.132341	1.259093	1.292891
**C**	-1.003591	-1.266255	-0.003858	H	-6.975536	2.500269	0.034295
**C**	-1.773251	-1.813224	1.021438	H	-7.774200	0.968129	-1.696650
**C**	-3.105880	-1.451987	1.160067	H	-7.581397	-0.424361	-0.610165
**C**	-3.676327	-0.523245	0.292581	H	9.834786	2.808745	-0.542083
**C**	-2.904818	0.017885	-0.738755	H	9.872301	2.473572	1.203961
**C**	-1.579142	-0.357288	-0.891684	H	11.127483	1.818724	0.145617
**C**	-5.111050	-0.172898	0.505779	H	9.782874	-1.164187	0.513807
**N**	-5.552061	1.003764	-0.026664	H	9.737701	-0.836481	-1.232629
**C**	-6.909094	1.431197	0.237393	H	11.077699	-0.230727	-0.249479
**C**	-7.853499	0.629415	-0.662258	H	-10.638655	0.038968	1.218083
**N**	-9.318667	0.703384	-0.292503	H	-9.130133	0.755377	1.827808
**N**	9.303167	0.830231	-0.004761	H	-9.102599	-0.874199	1.109584
**C**	10.065603	2.048945	0.212691	H	-11.143133	-0.052062	-1.060198
**C**	10.005170	-0.416940	-0.256347	H	-9.908166	0.326324	-2.294019
**C**	-9.562405	0.112074	1.065137	H	-9.734231	-1.127888	-1.273678
**C**	-10.082854	-0.096199	-1.305358	H	-9.618769	2.543663	-1.300610
**C**	-9.808340	2.117903	-0.315654	H	-10.877981	2.117815	-0.109298
**O**	-5.879742	-0.901149	1.127478	H	-9.289574	2.693298	0.449125

Table S5. The cartesian coordinates of molecule in the 2,2,2-trifluorpethanol solvent

Elements	X	Y	Z	Elements	X	Y	Z
**C**	7.280918	2.013440	0.280113	O	3.258711	-2.624996	-0.460310
**C**	7.961136	0.788525	0.008029	O	0.974124	0.817781	0.272973
**C**	7.188534	-0.374140	-0.186423	H	7.845151	2.922446	0.436029
**C**	5.813609	-0.299695	-0.112338	H	7.637374	-1.334821	-0.394059
**C**	5.131619	0.900129	0.152116	H	5.414520	2.997587	0.554785
**C**	5.914484	2.056415	0.347098	H	3.159021	1.767334	0.398196
**O**	5.120344	-1.453130	-0.309096	H	-1.442778	-2.592710	1.586826
**C**	3.744835	-1.535060	-0.267876	H	-3.836998	-1.950931	1.775828
**C**	3.026378	-0.299746	-0.000093	H	-3.280027	0.960437	-1.326789
**C**	3.722085	0.860136	0.199744	H	-0.929209	0.306574	-1.532202
**C**	1.551262	-0.229983	0.068234	H	-4.878060	1.846889	-0.207437
**S**	0.639577	-1.772778	-0.170437	H	-7.194843	1.283734	1.273375
**C**	-1.029633	-1.176019	0.023632	H	-7.006928	2.606562	0.112271
**C**	-1.851125	-1.812931	0.952928	H	-7.879873	1.315160	-1.706327
**C**	-3.186463	-1.453532	1.065675	H	-7.439905	-0.213721	-0.919033
**C**	-3.708729	-0.435191	0.271417	H	9.865078	2.720389	-0.597360
**C**	-2.884660	0.199019	-0.661922	H	9.949519	2.368687	1.143820
**C**	-1.556971	-0.174877	-0.792811	H	11.161195	1.703390	0.042324
**C**	-5.147999	-0.093959	0.443582	H	9.776743	-1.259640	0.432109
**N**	-5.559345	1.133817	0.003784	H	9.691701	-0.919820	-1.310528
**C**	-6.930454	1.526594	0.240818	H	11.066698	-0.342883	-0.359007
**C**	-7.835852	0.786475	-0.752841	H	-10.372507	-0.544887	1.093751
**N**	-9.266632	0.595233	-0.300037	H	-8.928549	0.240117	1.774446
**N**	9.317551	0.746036	-0.061787	H	-8.734788	-1.181458	0.728933
**C**	10.105554	1.949784	0.143491	H	-11.051755	-0.211945	-1.109506
**C**	9.991204	-0.510875	-0.338929	H	-9.963831	0.550389	-2.303705
**C**	-9.329538	-0.288274	0.913375	H	-9.554970	-1.044369	-1.610688
**C**	-10.014114	-0.076191	-1.413411	H	-9.866686	2.535633	-0.899997
**C**	-9.908959	1.914251	-0.005317	H	-10.946563	1.744807	0.280980
**O**	-5.957676	-0.869591	0.945649	H	-9.379747	2.399621	0.812758

Table S6. The cartesian coordinates of molecule in the nitroethane solvent

Elements	X	Y	Z	Elements	X	Y	Z
**C**	7.244308	2.059012	0.286233	O	3.312464	-2.646353	-0.516922
**C**	7.949601	0.844080	0.030016	O	0.959440	0.755861	0.200351
**C**	7.199067	-0.331732	-0.178536	H	7.791277	2.977186	0.449302
**C**	5.822664	-0.278327	-0.128594	H	7.668139	-1.284631	-0.377463
**C**	5.116282	0.910273	0.123528	H	5.357776	3.013324	0.526062
**C**	5.876886	2.080323	0.329398	H	3.129158	1.744408	0.346406
**O**	5.149326	-1.442976	-0.336456	H	-1.310419	-2.456418	1.761955
**C**	3.775985	-1.546493	-0.313821	H	-3.696181	-1.799307	2.001909
**C**	3.033696	-0.326130	-0.052067	H	-3.349579	0.668760	-1.490905
**C**	3.708558	0.846458	0.153764	H	-1.004400	0.005061	-1.737927
**C**	1.558084	-0.282543	0.008793	H	-4.907671	1.661657	-0.455457
**S**	0.670054	-1.842471	-0.208581	H	-7.129672	1.306130	1.254480
**C**	-1.002319	-1.257375	0.004145	H	-6.990661	2.490472	-0.059840
**C**	-1.761827	-1.768982	1.054769	H	-7.786062	0.866229	-1.713868
**C**	-3.092388	-1.400529	1.194955	H	-7.596136	-0.464826	-0.553503
**C**	-3.671375	-0.501177	0.302445	H	9.839028	2.803343	-0.542411
**C**	-2.909628	0.004668	-0.753706	H	9.880137	2.461536	1.202253
**C**	-1.585562	-0.376687	-0.906679	H	11.132116	1.809082	0.138693
**C**	-5.105507	-0.145806	0.518238	H	9.785274	-1.174952	0.492145
**N**	-5.560443	0.999343	-0.065600	H	9.735819	-0.835094	-1.251876
**C**	-6.917687	1.432040	0.190269	H	11.079030	-0.237519	-0.267910
**C**	-7.865449	0.585376	-0.662777	H	-10.642302	0.093858	1.253163
**N**	-9.329554	0.682510	-0.295883	H	-9.150502	0.877422	1.817012
**N**	9.306174	0.823424	-0.011109	H	-9.089965	-0.796251	1.204938
**C**	10.070755	2.040562	0.208998	H	-11.156871	-0.105315	-1.020906
**C**	10.006364	-0.422719	-0.273405	H	-9.911075	0.173588	-2.269900
**C**	-9.567444	0.176950	1.097076	H	-9.758571	-1.208429	-1.150152
**C**	-10.095806	-0.178166	-1.255590	H	-9.649768	2.448823	-1.422719
**C**	-9.822138	2.091644	-0.407721	H	-10.888595	2.103469	-0.185287
**O**	-5.860161	-0.847192	1.186868	H	-9.292700	2.717973	0.308116

Table S7. The cartesian coordinates of molecule in the water solvent

**Elements**	**X**	**Y**	**Z**	**Elements**	**X**	**Y**	**Z**
**C**	7.169600	2.091107	0.360733	O	3.343560	-2.668396	-0.640257
**C**	7.898907	0.922308	-0.024830	O	0.931233	0.602716	0.434127
**C**	7.174716	-0.261216	-0.295384	H	7.698027	3.010059	0.574248
**C**	5.804843	-0.253416	-0.179440	H	7.666647	-1.176622	-0.592711
**C**	5.072591	0.886860	0.198053	H	5.266975	2.958784	0.758437
**C**	5.807653	2.064370	0.465112	H	3.092747	1.649010	0.567811
**O**	5.148136	-1.423899	-0.449220	H	-1.133422	-2.266145	2.010544
**C**	3.779831	-1.562805	-0.373452	H	-3.484930	-1.571839	2.348531
**C**	3.018605	-0.396072	0.008122	H	-3.480620	0.293119	-1.521634
**C**	3.676550	0.780025	0.280820	H	-1.160186	-0.406211	-1.860580
**C**	1.552641	-0.399712	0.121047	H	-4.946743	1.410803	-0.548400
**S**	0.678621	-1.939900	-0.217428	H	-7.163057	1.241450	1.321807
**C**	-0.991808	-1.368705	0.058185	H	-6.949967	2.391174	-0.007496
**C**	-1.648842	-1.701709	1.240943	H	-7.579074	0.634619	-1.662682
**C**	-2.966530	-1.310780	1.433132	H	-7.717834	-0.561969	-0.356598
**C**	-3.634382	-0.570858	0.458506	H	9.688154	2.981531	-0.537753
**C**	-2.974464	-0.246926	-0.728611	H	9.840396	2.517289	1.170276
**C**	-1.661572	-0.648715	-0.929908	H	11.048656	1.984796	-0.006260
**C**	-5.054050	-0.184258	0.735407	H	9.797274	-1.069854	0.186250
**N**	-5.538737	0.892656	0.085144	H	9.680203	-0.587095	-1.518620
**C**	-6.907590	1.337524	0.266097	H	11.039379	-0.032704	-0.529106
**C**	-7.830336	0.492907	-0.610480	H	-10.876853	0.660364	0.906907
**N**	-9.301766	0.799523	-0.484265	H	-9.335953	1.224593	1.595729
**N**	9.247231	0.949572	-0.127068	H	-9.540607	-0.500619	1.164187
**C**	9.987264	2.175815	0.140470	H	-11.096797	0.110469	-1.356458
**C**	9.973013	-0.250245	-0.518360	H	-9.679018	0.112027	-2.447470
**C**	-9.795651	0.525488	0.900040	H	-9.822460	-1.131225	-1.167384
**C**	-10.029887	-0.095729	-1.436951	H	-9.183029	2.417492	-1.836818
**C**	-9.590086	2.221144	-0.844888	H	-10.671234	2.358780	-0.845756
**O**	-5.741804	-0.818607	1.544652	H	-9.136787	2.881132	-0.107437

Table S8. The cartesian coordinates of molecule without solvent

Elements	X	Y	Z	Elements	X	Y	Z
**C**	7.295140	1.994082	0.233850	O	3.219552	-2.614678	-0.391564
**C**	7.962697	0.743819	0.077469	O	0.968951	0.888137	0.032242
**C**	7.179036	-0.415540	-0.076869	H	7.868261	2.902850	0.355004
**C**	5.802539	-0.316839	-0.073391	H	7.616873	-1.395712	-0.199964
**C**	5.133176	0.909122	0.079960	H	5.436447	3.023281	0.354546
**C**	5.927538	2.062275	0.233643	H	3.163467	1.819862	0.186307
**O**	5.101648	-1.469778	-0.226744	H	-1.491046	-2.545093	1.480769
**C**	3.722666	-1.529304	-0.242823	H	-3.884636	-1.892473	1.635053
**C**	3.016547	-0.265518	-0.080958	H	-3.254495	1.032459	-1.446246
**C**	3.720362	0.894361	0.070238	H	-0.905403	0.377189	-1.602620
**C**	1.541547	-0.175873	-0.069774	H	-4.880610	1.904081	-0.420801
**S**	0.625866	-1.732650	-0.207129	H	-7.125077	1.498007	1.152271
**C**	-1.040139	-1.119463	-0.061868	H	-7.014797	2.676299	-0.163379
**C**	-1.885725	-1.760369	0.844264	H	-8.106313	1.248366	-1.715299
**C**	-3.220092	-1.396491	0.936910	H	-7.444499	-0.204094	-0.935191
**C**	-3.722490	-0.368569	0.140585	H	9.934951	2.596974	-0.567094
**C**	-2.876678	0.267360	-0.774069	H	9.924818	2.369135	1.196941
**C**	-1.549135	-0.109366	-0.881856	H	11.176609	1.607932	0.208962
**C**	-5.150099	-0.010981	0.304178	H	9.726368	-1.075651	-1.039697
**N**	-5.567374	1.200002	-0.197738	H	11.061439	-0.461886	-0.054838
**C**	-6.920909	1.617430	0.083805	H	9.713095	-1.301606	0.723453
**C**	-7.891326	0.771344	-0.756692	H	-10.040811	-0.670244	1.462207
**N**	-9.234030	0.493662	-0.109964	H	-8.742643	0.436531	1.964660
**N**	9.322483	0.674809	0.077717	H	-8.316849	-1.025515	1.062162
**C**	10.121607	1.876616	0.237684	H	-11.012088	-0.536641	-0.633910
**C**	9.982621	-0.608305	-0.081764	H	-10.101892	0.136633	-2.014479
**C**	-9.072180	-0.251470	1.189584	H	-9.500253	-1.319078	-1.172018
**C**	-10.019451	-0.368195	-1.051806	H	-10.140453	2.268116	-0.831010
**C**	-9.979604	1.768160	0.124862	H	-10.938807	1.536556	0.588945
**O**	-5.971483	-0.743779	0.852347	H	-9.398300	2.407889	0.788000

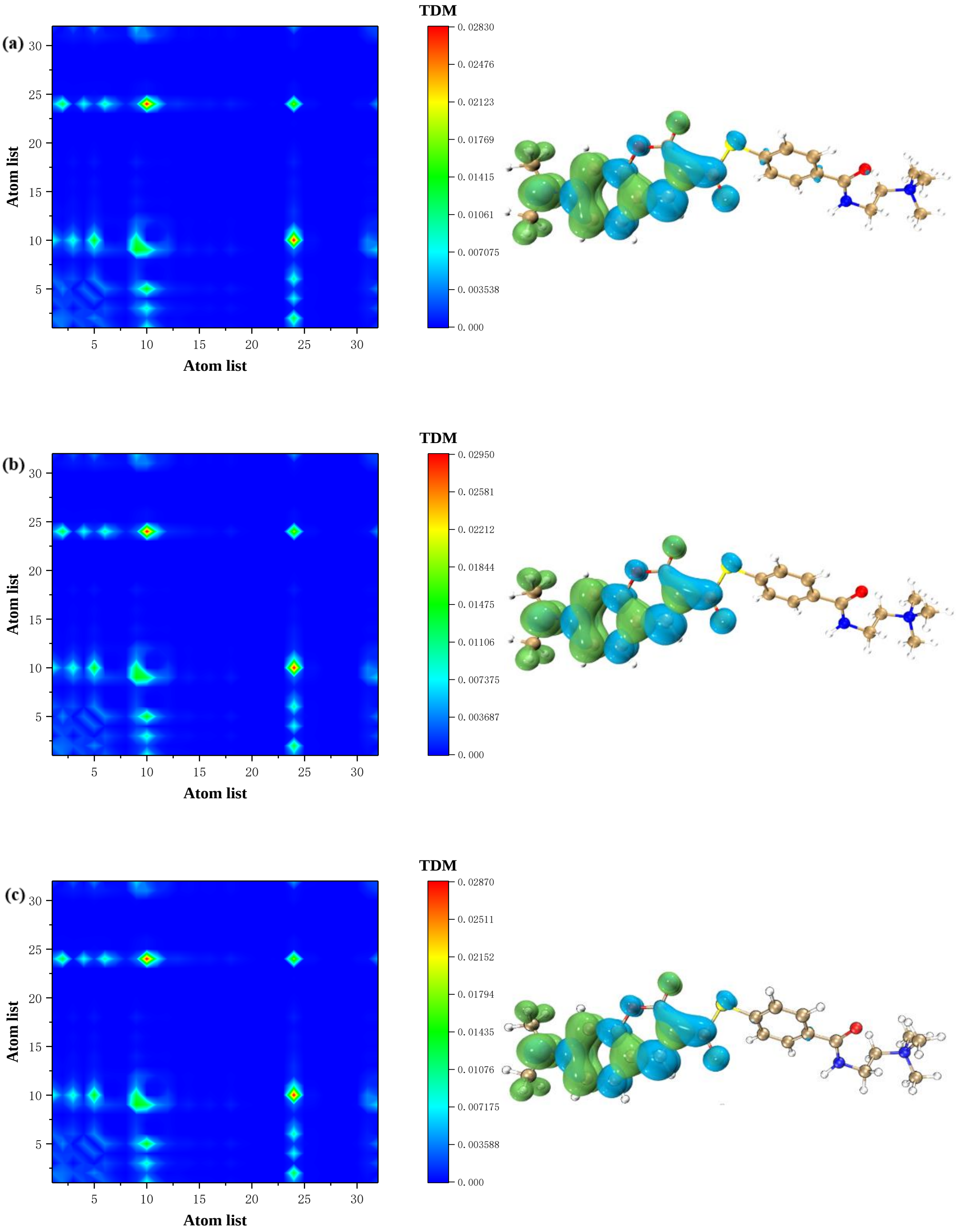
(a)
TDM
0.02830
0.02476
0.02123
0.01769
0.01415
0.01061
0.007075
0.003538
0.000
Atom list
Atom list
(b)
TDM
0.02950
0.02581
0.02212
0.01844
0.01475
0.01106
0.007375
0.003687
0.000
Atom list
Atom list
(c)
TDM
0.02870
0.02511
0.02152
0.01794
0.01435
0.01076
0.007175
0.003588
0.000
Atom list
Atom list

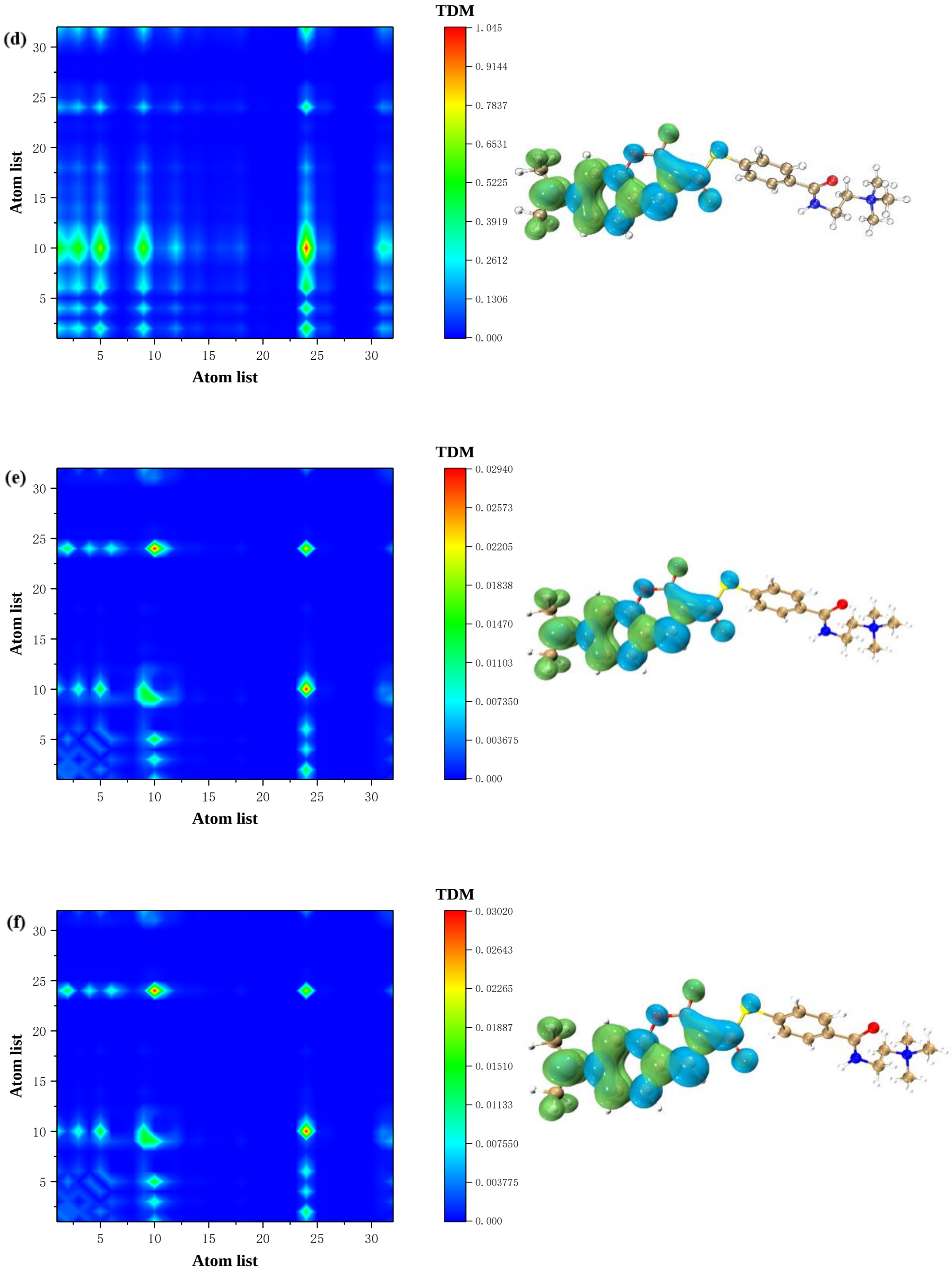

Figure S1. S_1 TDM and electron-hole pair analysis of molecule in p-isopropyltoluene (a), Thiophenol (b), Carbon disulfide (c), Diiodomethane (d), 2,2,2-trifluorpethanol (e) and Nitroethane (f).
